# Transcriptome sequencing revealed that knocking down *FOXL2* affected cell proliferation, the cell cycle, and DNA replication in chicken pre-ovulatory follicle cells

**DOI:** 10.1371/journal.pone.0234795

**Published:** 2020-07-09

**Authors:** Wei Luo, Lantao Gu, Jinqiu Li, Yanzhang Gong

**Affiliations:** 1 Key Laboratory of Agricultural Animal Genetics, Breeding and Reproduction of Ministry of Education, College of Animal Science and Technology, Huazhong Agricultural University, Wuhan, Hubei, China; 2 Guilin Medical University, Guilin, Guangxi, China; 3 Affiliated Hospital of Putian University, Putian, Fujian, China; Colorado State University, UNITED STATES

## Abstract

Forkhead box L2 (*FOXL2*) is a single-exon gene encoding a forkhead transcription factor, which is mainly expressed in the ovary, eyelids and the pituitary gland. *FOXL2* plays an essential role in ovarian development. To reveal the effects of *FOXL2* on the biological process and gene expression of ovarian granulosa cells (GCs), we established stable *FOXL2*-knockdown GCs and then analysed them using transcriptome sequencing. It was observed that knocking down *FOXL2* affected the biological processes of cell proliferation, DNA replication, and apoptosis and affected cell cycle progression. *FOXL2* knockdown promoted cell proliferation and DNA replication, decreased cell apoptosis, and promoted mitosis. In addition, by comparing the transcriptome after *FOXL2* knockdown, we found a series of DEGs (differentially expressed genes) and related pathways. These results indicated that, through mediating these genes and pathways, the FOXL2 might induce the cell proliferation, cycle, and DNA replication, and play a key role during ovarian development and maintenance.

## Introduction

As an animal with daily ovulation, a laying hen usually possesses 5–7 yellow follicles in the ovary concurrently based on a hierarchical sequence of pre-ovulatory follicles awaiting ovulation. One follicle is selected into the hierarchy from a cohort of pre-hierarchal follicles (small yellow follicles, SYF) after ovulation in a process termed follicle selection. Interactive communication among the oocyte, granulosa layer and theca layer is essential for the normal development of growing follicles. Ovarian granulosa cells (GCs) in the newly selected follicle initiates differentiation and becomes sensitive to gonadotrophins from the pituitary Moreover, major differences between GCs from pre-hierarchical (phGC) and pre-ovulatory follicles (poGC) lie in cell proliferation and steroidogenesis, for which the molecular basis remains unclear.

Forkhead box L2 (*FOXL2*) is a single-exon gene encoding a forkhead transcription factor, which is mainly expressed in the ovary, eyelids and the pituitary gland [1]. Many existing studies in humans, mice and other mammals have shown that *FOXL2* plays an essential role in ovarian development [2,3]. It has been established that *FOXL2* mutations are the cause of blepharophimosis, ptosis and epicanthus inversus syndrome (BPES), an autosomal dominant genetic disease in humans associated with premature ovarian failure (POF) [3,4]. Moreover, granulosa cells in *FOXL2*-mutation homozygous mice do not complete the squamous-to-cuboidal transition, leading to the absence of secondary follicles and oocyte atresia [5]. More than 95% of adult-type ovarian granulosa cell tumours (OGCTs) are highly associated with a somatic point mutation (C134W) in *FOXL2*, suggesting a potential relationship between *FOXL2* and human granulosa cell function [6]. Further studies in humans and mice indicate that the normal FOXL2 protein induces GC apoptosis and inhibits cell proliferation, while the mutant protein compromises these activities, thus contributing to OGCTs [7,8].

Although FOXL2 is highly conserved and participates in female ovarian development in various vertebrates, the exact functions of *FOXL2* differ among species [9]. For instance, *FOXL2* was reported to activate *CYP19A1* (the gene encoding aromatase) expression in human KGN cells [10,11] but repress *CYP19A1* in both Chinese hamster ovary cells [12] and murine primary GCs [13]. However, in a finding dramatically different than that for mammals, we recently discovered that *CYP19A1* is directly regulated by *SF1* (steroidogenic factor 1) and *ESR2* (estrogen receptor 2) instead of *FOXL2* in chicken GCs [14]. A previous study identified a novel SNP in *FOXL2* that is highly associated with egg production and egg weight in Chinese Dagu hens [15]. Another in vitro study showed that *FOXL2* facilitated the effect of members of the transforming growth factor beta (TGF-β) superfamily on follicle-stimulating hormone receptor (FSHR) expression and pre-hierarchical granulosa cell proliferation [16]. However, a systematic exploration of *FOXL2* function in chicken ovaries is needed.

To better understand the functions of *FOXL2* in chicken granulosa cells, we previously used high-throughput sequencing to analyse the transcriptomic changes induced by *FOXL2* overexpression and found that *FOXL2* exerted divergent roles in chicken pre-hierarchical cells (phGC) and pre-ovulatory granulosa cells (poGC) [14]. In the present study, another transcriptome analysis was performed for the case of *FOXL2* knockdown using RNA interference in both phGC and poGC. According to the results from the functional enrichment analysis of DEGs, we validated the differential effects of *FOXL2* on GC proliferation, DNA replication, apoptosis and the cell cycle in the phGC compared to the poGC.

## Materials and methods

### Animals and preparation

Sexually mature hens (25–30 weeks of age) with continuous laying performance were purchased from the Xinhua chicken farm (Hubei, China) and maintained in cages with available food and water. Four hens were killed by cervical dislocation, and follicles were selected according to three specific growth phases, and pre-hierarchical small yellow follicles (SYF, 6–8 mm in diameter) and pre-ovulatory F2-F4 follicles were detached [17,18]. All the hens involved in the study were housed and handled according to the recommendations in the Guide for the Care and Use of Laboratory Animals of the Ministry of Science and Technology of China and protocols approved by the Scientific Ethics Committee of Huazhong Agricultural University (permit number HZAUCH-2016-009). All efforts were made to minimize animal suffering.

### Granulosa cell culture

The primary granulosa cells were pre-cultured with Medium 199 (Gibco, USA) and 5% FBS (Gibco, USA) overnight (16 h) and transfected with FOXL2-specific siRNA (FOXL2-siRNA) or NC nonsense siRNA (NC-siRNA) using Lipofectamine 3000 (Invitrogen Life Technologies, Carlsbad, CA, USA) according to the manufacturer’s instructions. Small interfering RNA (siRNA) was purchased from RiboBio (Guangzhou, China). The siRNA sequences of FOXL2-siRNA are given in S1 Table (see the supplementary data section at the end of this article). Forty-eight hours after transfection, the cells were washed in PBS and collected for quantitative real-time PCR, Western blot and RNA-Seq analyses. Primary poGC and phGC transfected with FOXL2-siRNA or NC-siRNA were named poGC-KD, poGC-CT, phGC-KD and phGC-CT, respectively.

### Quantitative real-time PCR (qRT-PCR)

The same RNA samples from the NC-siRNA and FOXL2-siRNA groups used for RNA-Seq were subjected to qRT-PCR. Total RNA was extracted from primary granulosa cells using TRIzol reagent (Invitrogen, USA) following the manufacturer’s protocol. A Qubit RNA assay kit in a Qubit 2.0 A fluorometer (Life Technologies, CA, USA) was utilized to measure the RNA concentrations. The integrity of the obtained RNA was evaluated using an Agilent Bioanalyzer 2100 system (Agilent Technologies, CA, USA). High-quality total RNA was used for further experiments. RNA was reverse-transcribed using a PrimeScript^TM^ RT reagent kit with gDNA Eraser (TaKaRa, Japan), according to the instruction manual, and the obtained cDNA was stored at -20 ºC. qRT-PCR was carried out with a 10-μL volume of CFX-384 (Bio-Rad, USA) that included 5 μL of 2×iTaq^TM^ Universal SYBR Green SuperMix (Bio-Rad), 0.15 μL of 10 μM forward primer, and 0.15 μL of 10 μM reverse primer (S2 Table), and 4.7 μL of approximately 100 ng cDNA. The specificity of each primer was confirmed by investigating melting curves. Relative gene expression levels were calculated by the 2^-ΔΔCT^ [19] method, and the mean expression level of GAPDH was used as an internal control. Four biological duplicate samples were amplified in triplicate.

### Western blot analysis

The total protein sample of cells was extracted from primary granulosa cells using TRIzol reagent (Invitrogen, USA) according to the instruction manual, and the concentration was measured by BCA assays (TransGen, China). Each sample was separated by 12% SDS–PAGE gel and then transferred to a PVDF membrane. Then, the PVDF membrane was incubated and sealed with 5% skimmed milk powder (Biosharp, China) and 1% BSA (Biosharp, China) at room temperature for 1 h and then incubated with a diluted monoclonal anti-FOXL2 antibody (1:1000, Abcam, USA) or anti-GAPDH antibody (1:2000, Proteintech, USA) at 4°C overnight, followed by incubation with a secondary antibody (1:2000, Proteintech, USA) at room temperature for 2 h. After washing with TBST three times, protein localization was observed using ECL chemiluminescence reagent (Bio-Rad, USA) for 5 min, and the antigen-antibody complexes on the membranes were detected with an enhanced chemiluminescence (ECL) detection system (Bio-Rad, Hercules, CA, USA), and ImageJ was used for analyzing optical density to quantify signal intensity.

### Library preparation for RNA sequencing

The RNA library was prepared using a total amount of 1 μg RNA per sample. The sequencing library was generated under the instruction manual from the NEB Next Ultra^TM^ RNA Library prep Kit for Illumina (NEB, USA). The sixteen cDNA libraries that were obtained were sequenced on an Illumina HiSeq X-ten platform and generated paired-end reads.

### Analysis of the sequencing data

First, we removed some useless reads, which contained adapter, low quality reads and poly-N, from the raw data to obtain clean data. Furthermore, we calculated the content of GC (GC content), Q30 and sequence duplication level of the clean data.

Then, we mapped the high quality clean reads obtained to the Ensembl Gallus_gallus-5.0 (Gallus_gallus.Gallus_gallus-5.0.dna.toplevel.fa) database utilizing TopHat2. The level of gene expression was calculated by mapping the fragments per kilobase million (FPKM) of different samples. The calculation is as follows: FPKM = cDNA fragments / mapped fragments (millions) × transcript length (kb) [14].

### Differentially Expressed Genes (DEGs) and enrichment analysis

Two groups of DEGs (comp. poGC: poGC-KD vs. poGC-CT; comp. phGC: phGC-KD vs. phGC-CT) were compared utilizing the DESeq R package (1.10.1). DESeq supplies statistical routines for confirming the differential expression in digital gene expression data utilizing a model based on negative binomial distribution. DEGs were considered as genes with expression fold changes (FCs) > 1.5 and P-values < 0.05, as determined by DESeq. The DEGs were analysed with the DAVID server (https://david.ncifcrf.gov/) using the KEGG pathway database (http://www.kegg.jp/kegg/pathway.html) to obtain GO annotations and KEGG pathways. Fisher’s exact test was used to determine the significant GO categories and KEGG pathways. Items with corrected P-values less than 0.05 were considered to be significant or enriched.

### Cell proliferation assay

For the cell proliferation assay, we pre-cultured primary granulosa cells for 16 h in a 96-well plate at a density of 50,000 cells/well to guarantee cell viability before the treatment. Then, we transfected phGC and poGC with FOXL2-siRNA, NC-siRNA, or a blank (no transfection). Cell viability was evaluated utilizing a Cell Counting Kit-8 (CCK-8) following the manufacturer’s protocol (Dojindo, Japan). The absorbance of each well in the 96-well plate was measured with a microplate reader (Bio-Rad, USA) at 24 h, 48 h, and 72 h post-transfection. The final results were visualized and analysed using GraphPad Prism 7.0.

### EdU assay

For the cell DNA replication assay, we pre-cultured primary phGC and poGC for 16 h in a 96-well plate at a density of 50,000 cells/well. Then, we transfected the phGC and poGC with FOXL2-siRNA or NC-siRNA. Cell DNA replication was studied in each well utilizing a Cell Light EdU DNA imaging kit (RiboBio, China) following the manufacturer’s protocol. Briefly, fresh culture medium with 5-ethynyl-2’-deoxyuridine (10 μM EdU from the Cell Light EdU DNA imaging kit, Guangzhou RiboBio, China) was used as the replacement medium 6 h post-transfection and then cultured for 24 h. Finally, the cell nuclei were re-stained with Hoechst 33342 and observed under a fluorescence microscope (Eclipse, Nikon, Japan). EdU-positive cells were regarded as cell DNA replication-positive, the number of which were calculated as (EdU add-in cells/Hoechst stained cells) ×100%.

### Flow cytometry detection of the cell cycle phases and apoptosis

In cell cycle assays and cell apoptosis assays, we pre-cultured primary granulosa cells for 16 h in a 12-well plate at a density of 500,000 cells/well. Then, we transfected phGC and poGC with FOXL2-siRNA or NC-siRNA. The cells from each well were harvested 48 h post-transfection, and the cell samples were collected and stained with a propidium iodide (PI) solution and then subjected to cell cycle assessment and analysis with a Cytomics FC 500 cytometer (Beckman, USA). The final results were analysed using ModFit LT software. The percentage of apoptotic cells was measured by double staining with Annexin V/ PI. In brief, we added 100 μL of binding buffer containing 1 μL of PI and 2.5 μL of Annexin V-FITC to the cell suspension and incubated the cells in the dark for 30 min. Then, the samples were analysed using Cytomics FC 500 to obtain the cell apoptosis rate.

### Statistical analysis

All data were processed following the individual procedures described above. Paired comparisons were evaluated using two-tailed Student’s *t*-test in Microsoft Excel 2016, and the significance was numerically expressed in figures (* indicates P < 0.05, ** indicates P < 0.01, *** indicates P < 0.001, ns indicates not significant). Multiple comparisons were performed and assessed using Duncan’s post hoc test in SPSS statistics software (version 23.0 for Mac, IBM, USA). Differences for which P < 0.05 were considered significant. All numerical results were visualized utilizing GraphPad Prism 7.0 (GraphPad software, USA) [20].

## Results

### RNA interference of *FOXL2* expression in chicken follicular GCs

**Fig 1:**
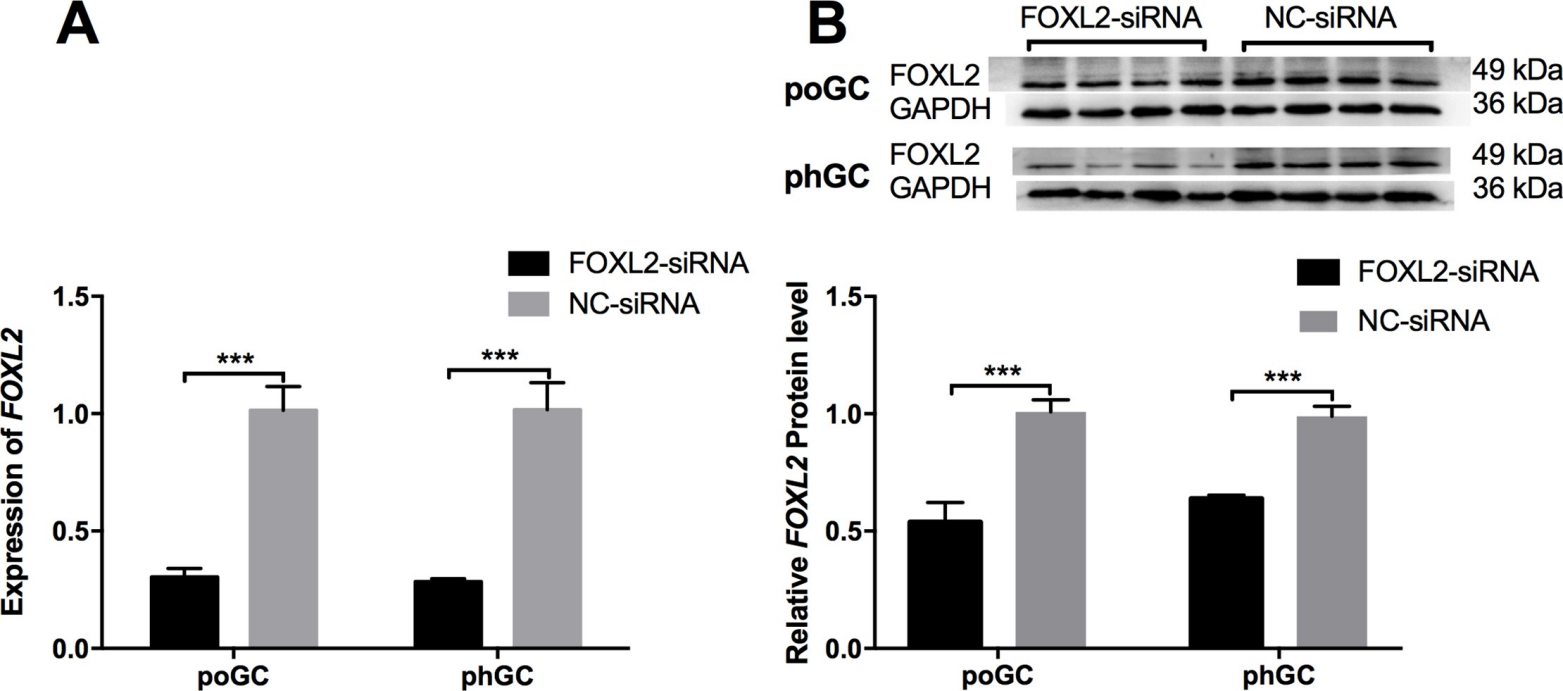
RNA interference of the *FOXL2* gene expression in the GCs. (A) poGC and phGC were transfected with FOXL2-siRNA or NC-siRNA, and the expression of the *FOXL2* gene was detected by qRT-PCR 48 h post-transfection. (B) poGC and phGC were transfected with FOXL2-siRNA or NC-siRNA, and the expression of the FOXL2 protein was detected by Western blot 48 h post-transfection (*** indicates P < 0.001).

To determine the effect of RNA interference of *FOXL2* expression in chicken follicular GCs, primary phGC and poGC were cultured before FOXL2-siRNA transfection. The qRT-PCR results showed that the expression levels of *FOXL2* in the poGC-KD and phGC-KD groups were significantly decreased (P < 0.001) compared with the those in the corresponding poGC-CT and phGC-CT groups (Fig 1A), findings consistent with the changes in *FOXL2* at the protein level as revealed by Western blot analysis results (Fig 1B). The results described above showed that FOXL2-siRNA successfully knocked down both the mRNA and protein expression of *FOXL2* in both the poGC and phGC. Full-length gels and Western blots are included in the S1 Raw images.

### RNA-Seq and Differentially Expressed Genes (DEGs)

To obtain a global view of the role of FOXL2 in chicken granulosa cells, we performed comparative transcriptomic analyses between FOXL2-siRNA- and NC-siRNA-treated groups of phGC and poGC. Sixteen cDNA libraries from four groups (poGC-KD, poGC-CT, phGC-KD and phGC-CT, 4 duplicates in each group) were established and sequenced. After sequencing quality control was performed, approximately 478.7 million clean reads (raw paired-end reads) were obtained, and each sample yielded approximately 29.9 million high-quality clean reads, ranging from 22.9 to 36.2 million. Subsequently, 83.4–86.1% of the reads were successfully mapped to the *Gallus gallus* genome (S3 Table).

**Fig 2:**
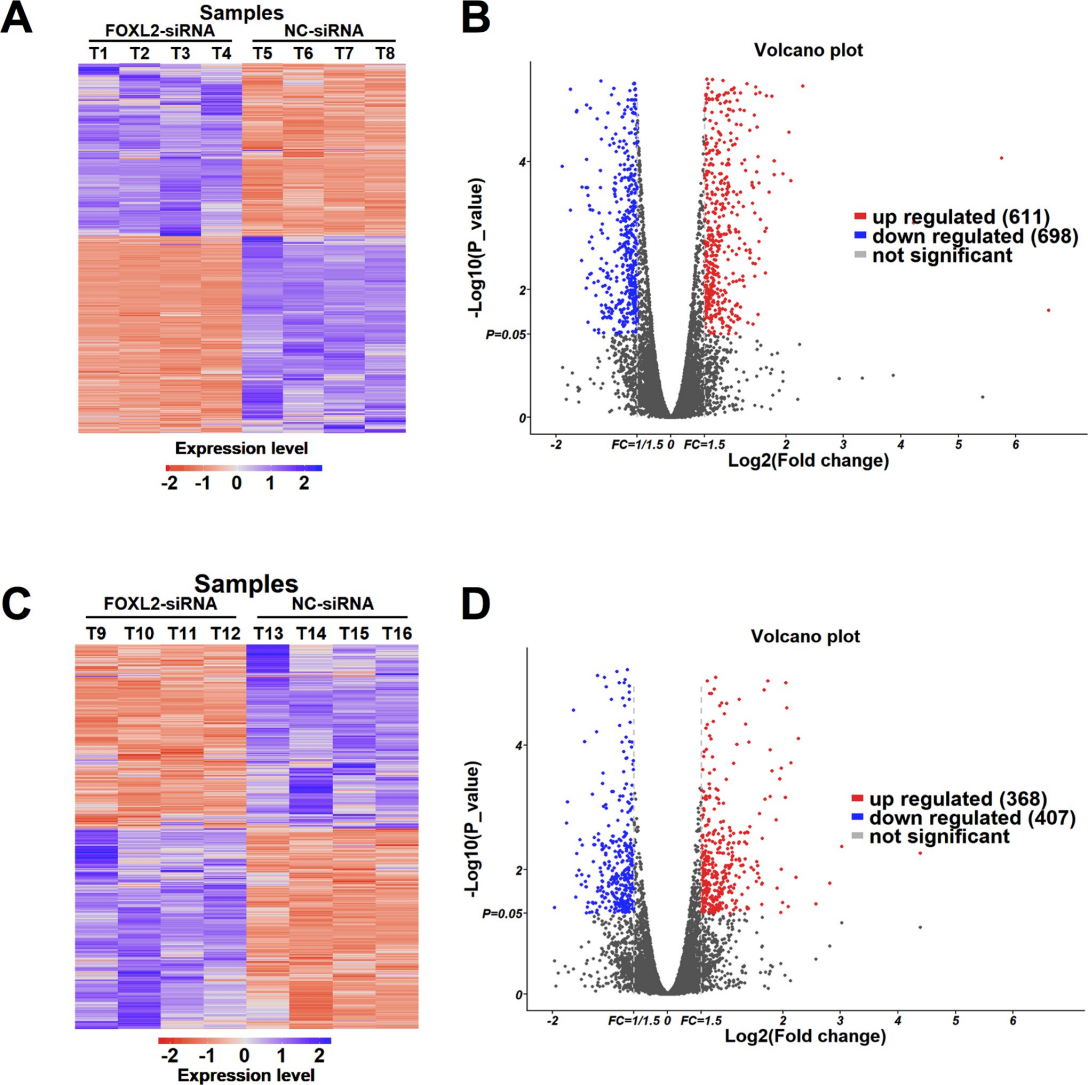
The expression patterns of the DEGs in the chicken granulosa cells induced by FOXL2-siRNA and NC-siRNA. (A) The hierarchical clustering of differentially expressed mRNAs of Comp. po; (B) The Volcano plot of differentially expressed mRNAs of Comp. po; (C) The hierarchical clustering of differentially expressed mRNAs of Comp. ph; (D) The Volcano plot of differentially expressed mRNAs of Comp. ph.

To obtain DEGs, pairwise comparisons were performed as follows: poGC-CT (poGC transfected with NC-siRNA) vs. poGC-KD (poGC transfected with FOXL2-siRNA) (denoted as comp. poGC) and phGC-CT (phGC transfected with NC-siRNA) vs. phGC-KD (phGC transfected with FOXL2-siRNA in pre-hierarchical granulosa cells) (denoted as comp. phGC). According to the criteria in which the fold change > 1.5 and P-value < 0.05, the results from the FPKM analysis showed that a total of 1309 and 775 DEGs were identified among the comp. poGC and comp. phGC, respectively, among which 611 genes were upregulated, and 698 genes were downregulated in the comp. poGC group, and 368 genes were upregulated and 407 genes were downregulated in comp. phGC group. Their distinct expression patterns are presented in the hierarchical clustering analysis and volcano plot (Fig 2).

### Experimental validation of the DEGs by qPCR

**Fig 3:**
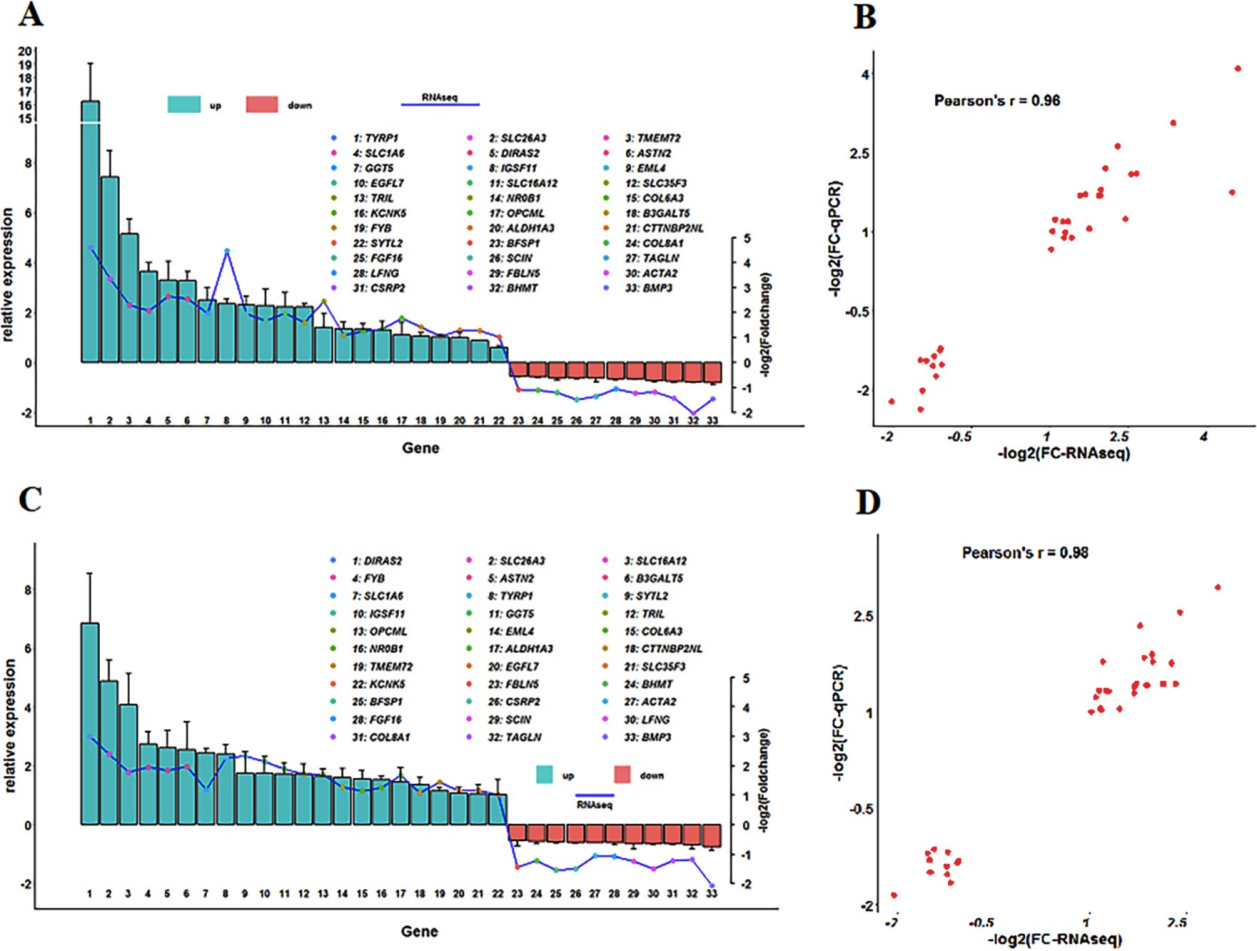
Experimental validation of the DEGs by qRT-PCR and correlation analysis of the qRT-PCR and RNA-Seq data. (A, C) The relative expression levels of the DEGs according to qRT-PCR (green or orange bars) were compared with transcripts determined from the RNA-Seq (blue line) data based on comp. poGC and comp. phGC. (B, D) A scatter plot of the qRT-PCR data and RNA-Seq data based on comp. poGC and comp. phGC.

To evaluate the reliability of the RNA-Seq data, 33 genes were randomly selected from the in different groups, and the expression pattern of each was verified by qRT-PCR. As shown in Fig 3A and Fig 3C, the mRNA expression levels of the 33 selected genes were highly consistent with the results of the DEG analysis of the sequencing data, and the qPCR results were significantly and positively correlated with the RNA-Seq data, as confirmed by a Pearson correlation analysis (comp. poGC: Pearson’s r = 0.96, P = 0; comp. phGC: Pearson’s r = 0.98, P = 0), revealing the high accuracy and quality of the RNA-Seq analysis data (Fig 3B and Fig 3D).

### GO and KEGG analyses of the DEGs

**Fig 4:**
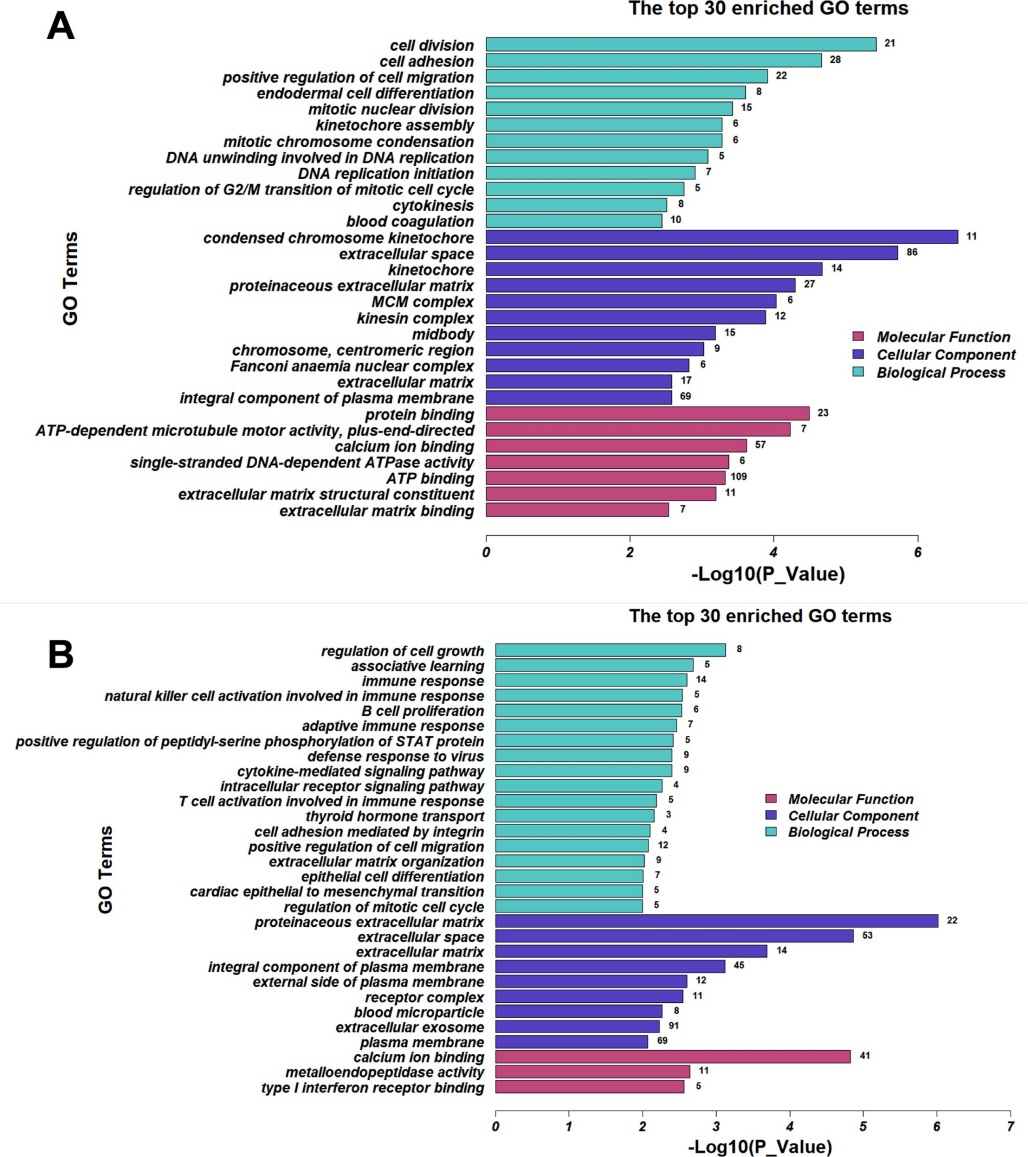
The most enriched Gene Ontology (GO) terms associated with the DEGs in the chicken granulosa cells induced by *FOXL2* silencing. (A) GO terms of DEGs in the comp. poGC; (B) GO terms of DEGs in the comp. phGC. The red, violet and blue bars represent the terms of molecular function, cellular component and biological process, respectively.

Gene Ontology (GO) was used to analyse the DEGs obtained from the comp. phGC and comp. poGC groups. According to three main ontologies, the top 30 GO terms for the most-enriched genes were summarized as biological process, cellular component and molecular function (Fig 4A and Fig 4B).

For the comp. poGC group, the top six GO term categories in which the differentially expressed genes were most enriched were (1) cell division, (2) cell adhesion, (3) mitotic nuclear division, (4) condensed chromosome kinetochore, (5) kinetochore and (6) MCM complex. And in the meanwhile, the top five GO term categories, which the differentially expressed genes were most enriched in, were (1) regulation of cell growth, (2) associative learning, (3) immune response, (4) proteinaceous extracellular matrix and (5) extracellular space in Comp. phGC.

**Fig 5:**
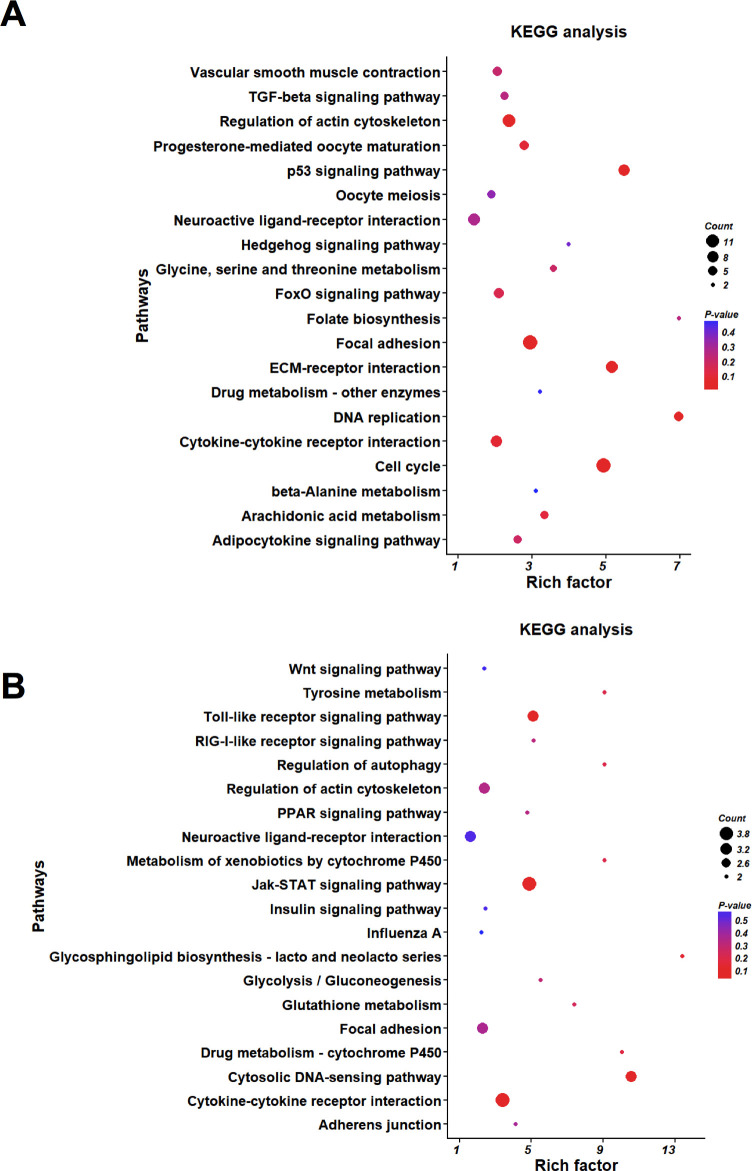
KEGG analysis of the DEGs induced by *FOXL2* silencing in the chicken granulosa cells. (A) KEGG enrichment analysis of DEGs in the comp. poGC. (B) KEGG enrichment analysis of DEGs in the comp. phGC. The 20 pathways in which the top DEGs were found are summarized. The Y-axis and X-axis indicate functional pathways and the enrich factor for the DEGs in the same pathway, respectively.

A KEGG pathway analysis was performed, and the top 20 pathways with the most-enriched DEGs are shown in Fig 5A and Fig 5B. The differentially expressed genes in comp. poGC group were enriched in pathways (P < 0.05) that included the cell cycle (gga04110), ECM-receptor interaction (gga04512), focal adhesion (gga04510), DNA replication (gga03030), the p53 signalling pathway (gga04115), etc. The differentially expressed genes in the comp. phGC group were enriched in pathways (P < 0.05) that included ECM-receptor interaction (gga04512), focal adhesion (gga04510), cytokine-cytokine receptor interaction (gga04060) and the Jak-STAT signalling pathway (gga04630). The details of the DEGs revealed in the GO and KEGG enrichment analyses are listed in the S1 File. And we compared the results of previous overexpression with our knockdown results, and screened out DEGs which were affected by both overexpression and knockdown of FOXL2. The details of those DEGs were listed in S2 File.

### *FOXL2* knockdown promotes primary poGC proliferation

**Fig 6:**
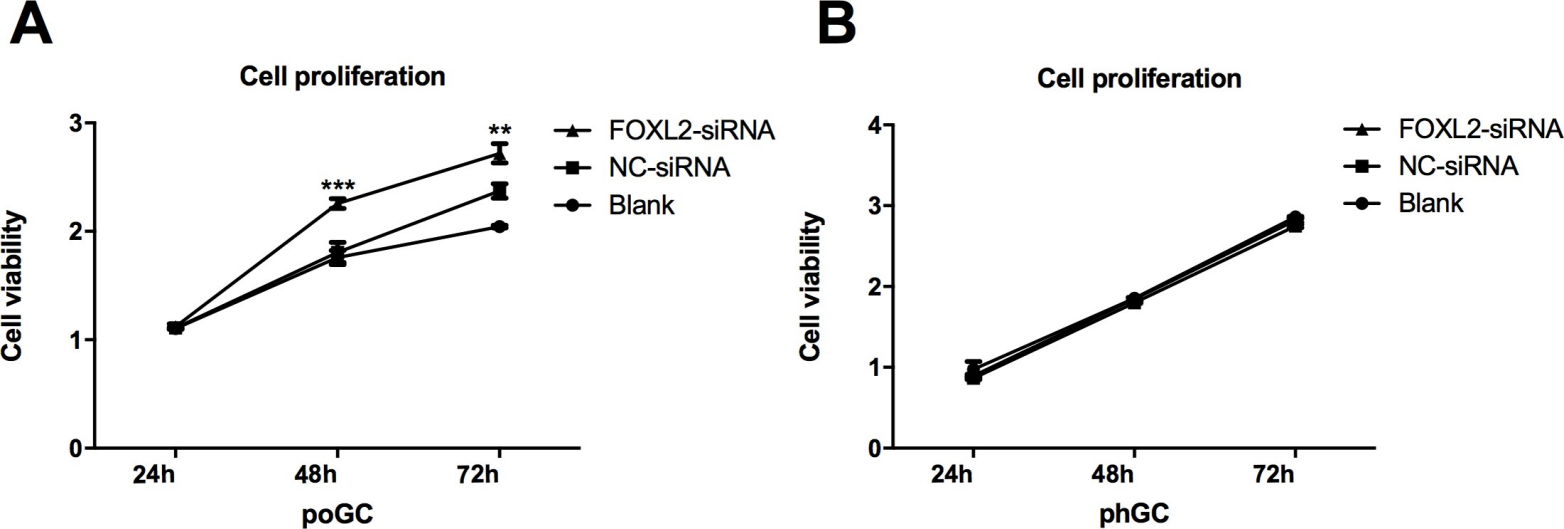
The effect of *FOXL2* knockdown on chicken granulosa cell proliferation. Chicken granulosa cells were transfected with FOXL2-siRNA, and two control groups were used: NC-siRNA and a blank (not transfected). Cell proliferation was measured using Cell Counting Kit-8 24, 48, and 72 h post-transfection. All values are represented as the mean ± SD of three independent experiments repeated in triplicate. The asterisks represent statistically significant differences (** indicates P < 0.01, *** indicates P < 0.001). (A) FOXL2-silencing in poGC promoted cell proliferation. (B) FOXL2-silencing in phGC had no effect on cell proliferation.

Cell proliferation capacity was measured with a CCK-8 assay, and we observed a profound and significant increase in cell proliferation in the poGC-KD group 48 h and 72 h post-transfection compared to increase in the poGC-CT and in the blank groups (P < 0.001) (Fig 6A). However, there was no significant difference in cell viability between the phGC-KD and phGC-CT groups and the blank group (Fig 6B). These results suggested that FOXL2 inhibited cell proliferation in the poGC, while no significant effect was observed in the phGC.

### FOXL2 knockdown promotes DNA synthesis in the poGC

**Fig 7:**
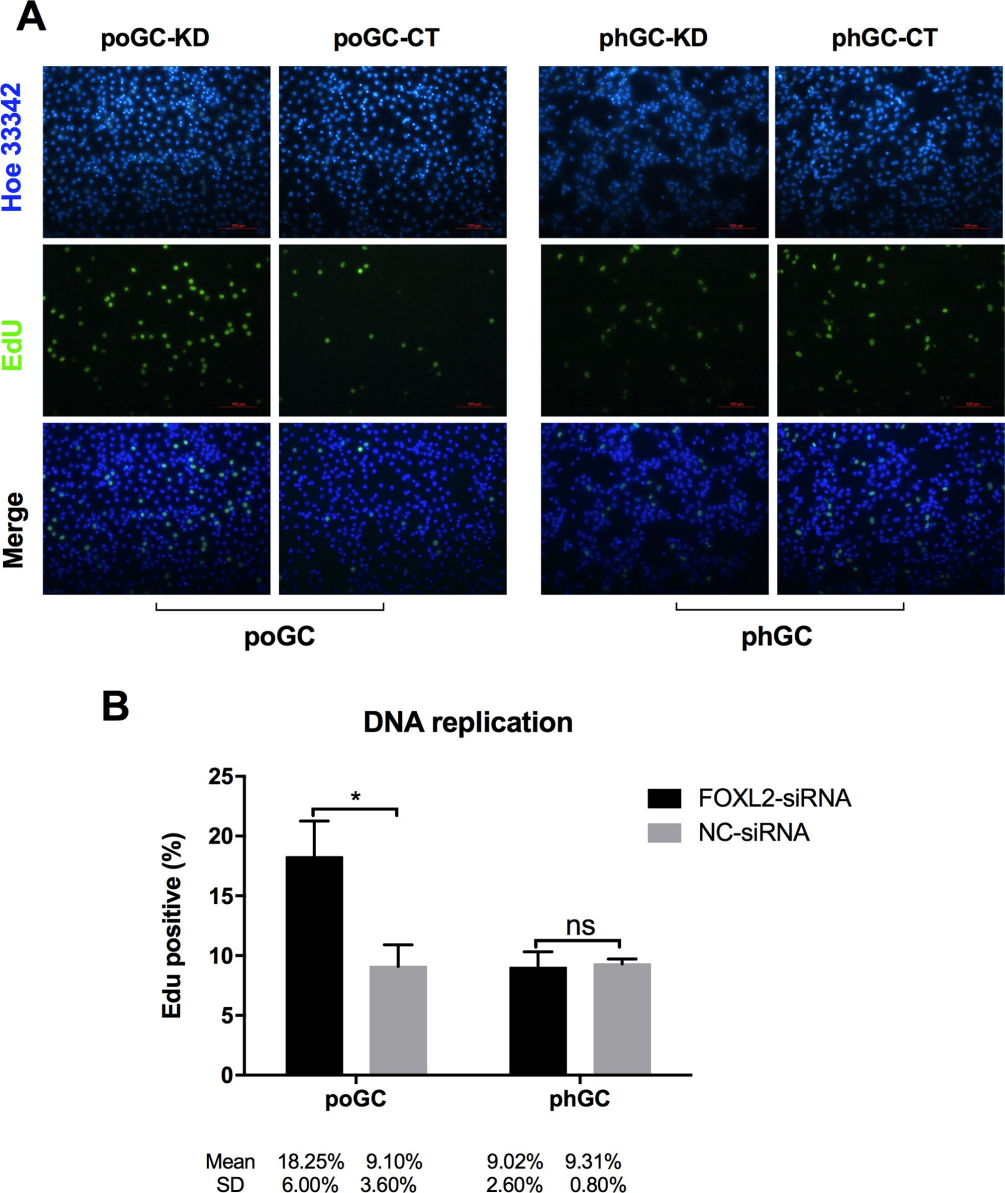
The effect of knocking down *FOXL2* on DNA replication in the chicken granulosa cells. (A) Representative images of Hoechst 33342-stained cells (top, cell nuclei), EdU-stained cells (middle, DNA replication) and the merged images (bottom) of the four groups (poGC-KD, poGC-CT, phGC-KD and phGC-CT). Primary phGC and poGC were transfected with FOXL2-siRNA or NC-siRNA, and fresh EdU-containing medium replaced the original medium 6 h post-transfection. Then, the cells were cultured for an additional 24 h. (B) Summary of EdU add-in cells as percentages of the granulosa cells. Data are presented as the mean ± SD (n = 4) (* indicates P < 0.05; ns indicates not significant).

An EdU assay was conducted to determine whether knocking down *FOXL2* had an impact on the DNA replication in the GCs. The EdU add-in cells represent cells that have active DNA synthesis, which indicates that they are predominantly in the S phase of the cell cycle. The results showed that the poGC-KD group (18.25 ± 6.02%) had more active DNA synthesis than the control poGC-CT group (9.10 ± 3.6%). However, there was no significant effect on the phGC, with EdU-positive percentages of 9.03 ± 2.64% and 9.31 ± 0.83% in the phGC-KD and phGC-CT groups, respectively (Fig 7).

### *FOXL2* knockdown inhibits cell apoptosis in the poGC

**Fig 8:**
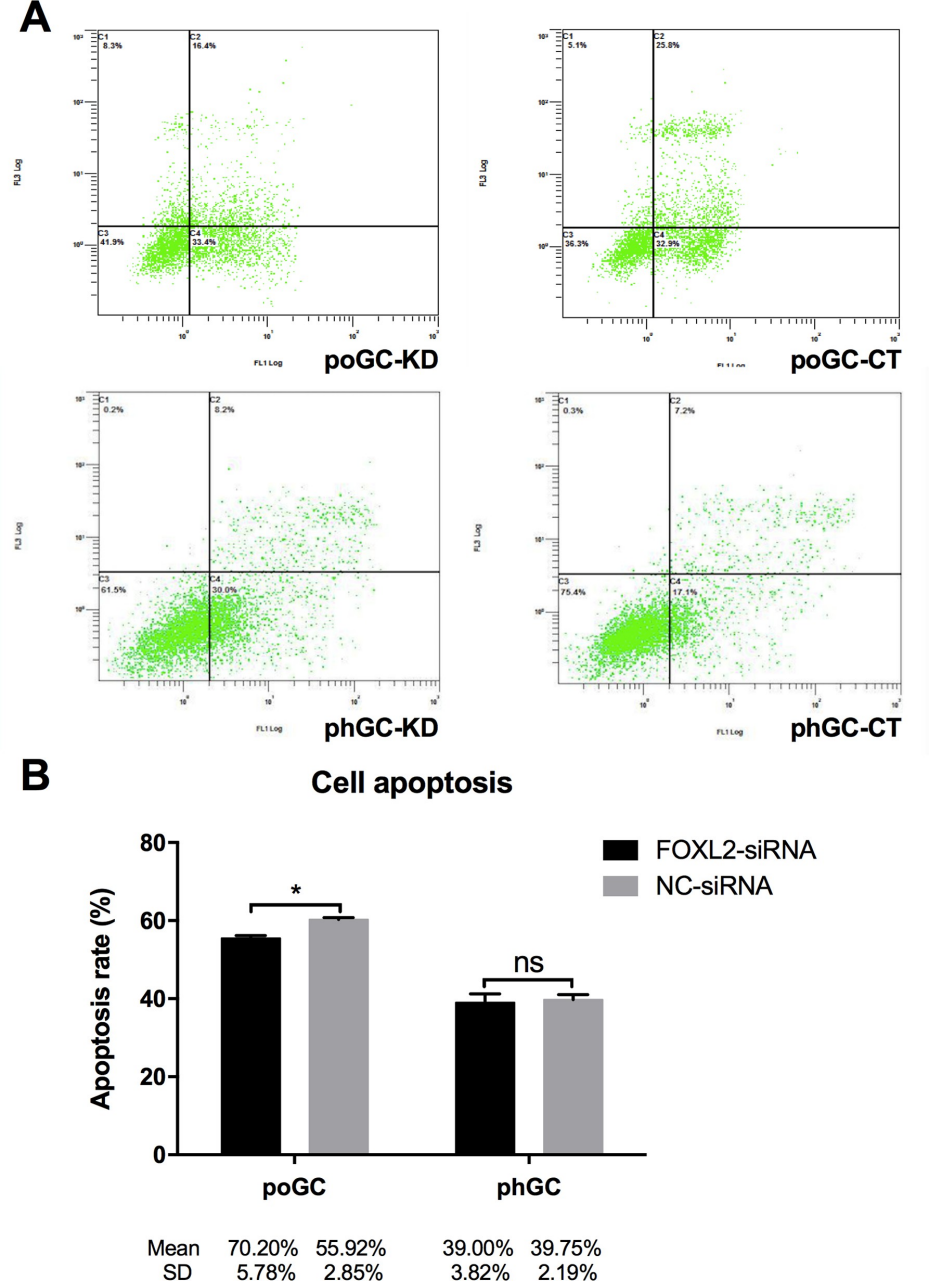
The effect of knocking down *FOXL2* on chicken granulosa cell apoptosis. poGC and phGC were transfected with FOXL2-siRNA or NC-siRNA, and the number of cells undergoing apoptosis was determined using a flow cytometer 48 h post-transfection. (A) Cell apoptosis of the groups (poGC-KD, poGC-CT, phGC-KD and phGC-CT) was determined by flow cytometry. (B) Apoptotic cells (Annexin V+/PI- and Annexin V+/PI+) are presented as the mean ± SD (n = 3). Two-tailed Student’s t-tests were used to analyse significant differences (* indicates P < 0.05; ns indicates not significant).

Cell apoptosis of the poGC-KD, poGC-CT, phGC-KD and phGC-CT was detected by flow cytometry using the Annexin V-PI staining method, and the results showed that the apoptosis rate of the poGC-KD group (55.52 ± 1.12%) was significantly less than that of the poGC-CT group (60.30 ± 0.77%). However, knocking down *FOXL2* had no significant effect on phGC apoptosis, with apoptosis rates of 36.65 ± 3.74% and 38.15 ± 5.43% for the phGC-KD and phGC-CT groups, respectively. It is noteworthy that the poGC had a higher rate of apoptosis than the phGC (Fig 8).

### *FOXL2* knockdown prevents cell cycle progression of the primary poGC

**Fig 9:**
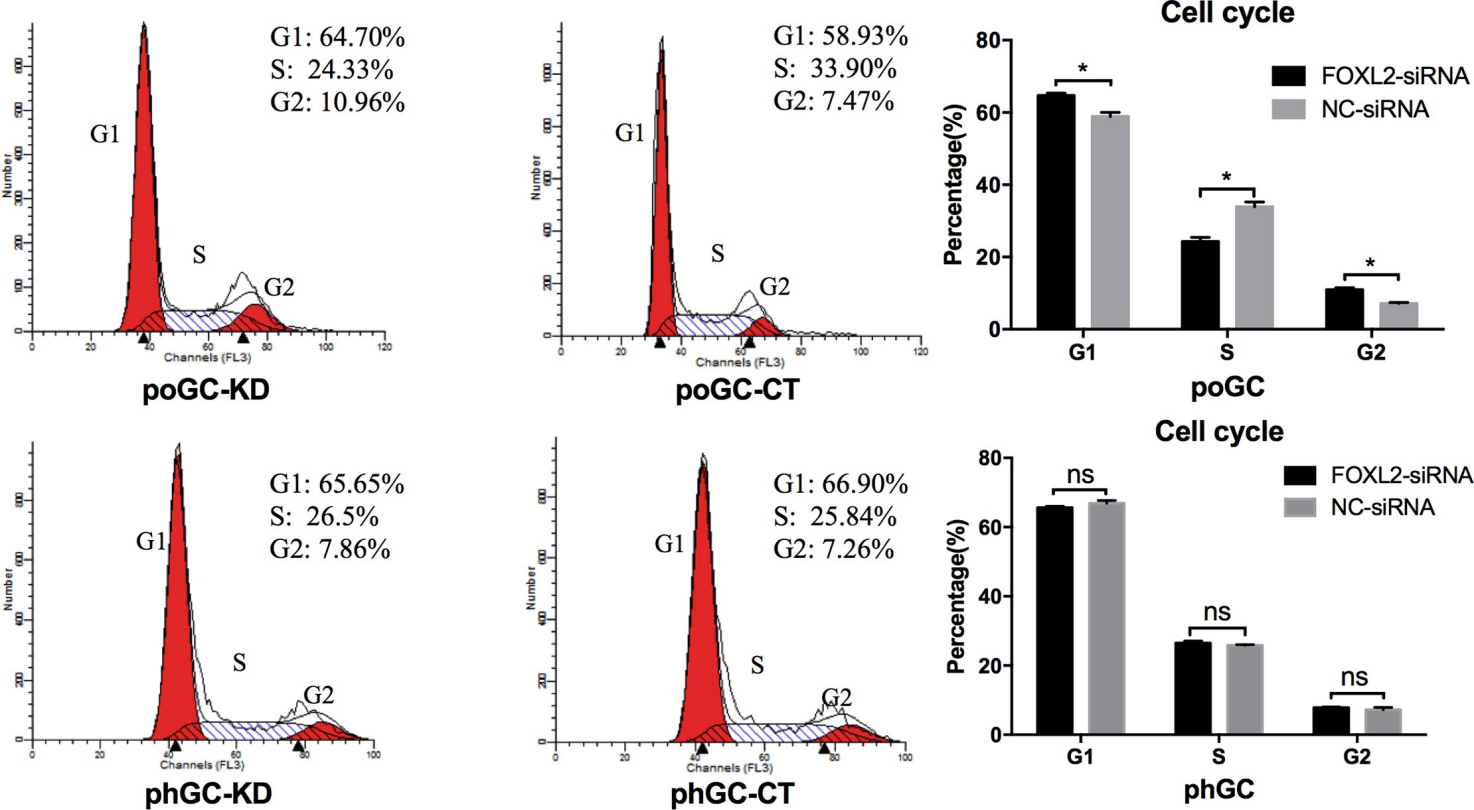
The effect of knocking down *FOXL2* on the cell cycle in chicken granulosa cells. PoGC and phGC were transfected with FOXL2-siRNA or NC-siRNA, and the distribution of cells by cell cycle phase was analysed using a flow cytometer 48 h post-transfection. Three independent experiments were repeated in triplicate. All the values are presented as the mean ± SD. The asterisks represent statistically significant differences (* indicates P < 0.05; ns indicates not significant).

To investigate the influence of *FOXL2* knockdown on cell cycle progression, the percentage of cells in each cycle phase were quantified for the 4 primary GC groups (poGC-KD, poGC-CT, phGC-KD and phGC-CT) by a flow cytometer. The proportion of cells in the G2 phase in poGC-KD and poGC-CT groups was 10.96% and 7.47%, respectively, and the difference was significant (P < 0.05). There was no significant difference in the cells in the G1 phase of the cell cycle for the phGC-KD (7.86%) and phGC-CT (7.26%) groups. The results show that the percentage of cells in the G2 phase in the FOXL2-silencing group (poGC-KD) was significantly increased compared to that of the control group (poGC-CT). Flow cytometry showed that FOXL2 silencing promoted mitosis of the poGC by inducing their transition into the G2 phase, but had no significant effect on the cell cycle progression of the phGC (Fig 9).

## Discussion

In a previous study, we explored the roles of chicken *FOXL2* that was overexpressed in GCs, and the results from the RNA sequencing showed that *FOXL2* exerted differential functions in poGC and phGC. To confirm the difference in *FOXL2* function between these two stages of GCs, we used RNA-interfering technology to knock down *FOXL2* expression in both poGC and phGC and then performed transcriptome analysis to show the *FOXL2* functions in terms of GC biology. Results from the transcriptome analysis showed that the expression of 1309 genes in the poGC and 775 genes in the phGC (267 genes were common to both) were significantly changed following *FOXL2* knockdown. Then, 33 randomly selected genes were subjected to qPCR, and the results were highly consistent with the DEGs determined based on the RNA-Seq data, validating our DEG analysis. It seems that FOXL2 plays a role in the transcriptional regulation of these genes directly or indirectly. Indeed, a portion of the potential transcription targets of FOXL2 have been reported to date. The gene encoding *StAR* involved in intramitochondrial cholesterol transport was transcriptionally repressed by *FOXL2* in human cells [10,21]. The expression of *CYP17A1* gene which plays a very important role in the synthesis of sex hormones, and encodes an enzyme responsible for estrogen production or androgen synthesis was also regulated by *FOXL2* in several mammalian models, including goat and mouse ovaries and human cells[22]. In addition, *FOXL2* was reportedly required for the transcription of *FST*[23], and the deletion of *FOXL2* might reduce *FST* expression in proliferating GCs, thus causing premature depletion of the ovarian reserve and inhibited formation of Sertoli-like cells in the ovary [24,25]. As previously described, other potential *FOXL2* transcription targets include *SOX9*, *CYP17A1*, *HAS2*, *FAS*, *PTGS2*, *BCL2A1* and so on. Among these and other genes, *FST*, *CYP19A1*, *FAS*, *PTGS2* and *BCL2A1* were reportedly activated by *FOXL2*, and *SOX9*, *CYP17A1* and *HAS2* were repressed by *FOXL2* [26]. Our transcriptome analysis results of these gene were also coincident with this expression pattern in chicken follicle. It furtherly confirmed the markable regulatory role of FOXL2 on these genes.

GO and KEGG pathway analyses were performed to characterize the mechanisms of *FOXL2* action in both the poGC and phGC. As shown in Figs 4 and 5, GO and KEGG enrichment varied between comp. poGC and comp. phGC, indicating different molecular mechanisms of *FOXL2* in these two stages of GCs. Therefore, although both *FOXL2* interference affected them both, the effect was not the same as it was for the gene expression patterns of the two types of granulosa cells (poGC and phGC). Since the genes, such as the epigenetic, hormone or cytokine factors, with expression patterns varied between the two kinds of cells following *FOXL2* interference, Wang suggested that *FOXL2* plays different roles at these two stages of chicken granulosa cell development: (1) promoting follicle selection in pre-hierarchical granulosa cells and (2) suppressing ovulation in pre-ovulatory granulosa cells [14]. Thus, GO and KEGG pathway analyses were performed to characterize the mechanisms of FOXL2. Many of the differentially expressed genes were annotated to biological functions, such as the TGF-beta pathway, oocyte meiosis, ECM-receptor interaction, cell cycle, p53 signalling pathway, and progesterone-mediated oocyte maturation. These biological processes directly or indirectly involve cell proliferation, differentiation, folliculogenesis and ovarian development. As already noted, the TGF-beta pathway can be promoted by *FOXL2* to influence GC proliferation and ovarian development [25]. The response to GO terms and KEGG pathways were enriched in our data. Many genes affecting cell biological functions were changed in Comp.po, such as minichromosome maintenance proteins (MCM) and cyclin-dependent kinases (CDK). MCM is one of the important regulators of eukaryotic DNA replication, not only regulates the initiation and elongation of DNA replication, but also plays an important role in DNA transcription and repair during meiosis and mitosis. And these genes are essential factors for normal ovarian development[27,28]. Research has shown that small, growing oocytes in mouse are not competent to mature into fertilizable eggs because they do not possess adequate amounts of cell cycle regulatory proteins, particularly cyclin-dependent kinase 1 (CDK1) [29]. As oocytes grow, they synthesize CDK1 and acquire the ability to mature. Follicle rupture during ovulation requires extracellular matrix (ECM) degradation at the apex of the follicle[30]. The p53 signalling pathway is involved in apoptosis, cell cycle progression and cell growth [31].

In a previous study, *FOXL2* was extensively reported to play an central role in essentially all stages of ovarian development and function, as well as in the lifetime maintenance of GC identity [32]. The overexpression of *FOXL2* was found to inhibit cell cycle progression at G1/S, thus interfering with cell proliferation and inducing apoptosis in granulosa cells [31]. Whether the situation is reversed during foxl2 expression interference remains to be studied?

Considering that many different functions enriched with DEGs in the comp. poGC group, but not in comp. phGC group, are associated with cell division, DNA replication, cell cycle and mitotic activity, we conducted further experiments to evaluate the effects of *FOXL2* on cell proliferation, DNA replication, apoptosis and cell cycle progression. Thus, we further examined cell proliferation, DNA replication, apoptosis, and the number of cells in each cell cycle phase by experiments designed to determine the effect of *FOXL2* knockdown in the poGC and phGC. The results showed that the cell viability in poGC-KD group was profoundly and significantly higher than that of the poGC-KD group 48 h post-transfection. The results showed that the poGC-KD group had cells with more active DNA synthesis than those in the control poGC-CT group. In addition, the apoptosis rate for the poGC-KD group was less than that of the poGC-CT group. The proportion of poGC-KD in the G2 phase was significantly higher than that of poGC-CT. In contrast to these measures during overexpression, *FOXL2* silencing promoted cell proliferation, mitosis, apoptosis and DNA synthesis in the poGC. These results further confirmed the notable role of *FOXL2* in regulating cell cycle progression, cell proliferation, apoptosis and DNA replication in poGC. The results from the CCK-8 assays showed that *FOXL2* inhibited poGC proliferation in a manner that resembled the role of *FOXL2* in human cervical cancer cells [33]. The results from the EdU assay indicated an inhibitory effect of *FOXL2* on the DNA synthesis of the poGC, a finding that was consistent with the CCK-8 results. In addition to the many studies on mammal granulosa cells have shown role of *FOXL2* in apoptosis [2,34,35], we also observed that chicken *FOXL2* knockdown decreased the cell apoptosis rate in the poGC. The role of FOXL2-regulation in the cell cycle in granulosa cells has also been published [7,31,34].

The results consistently revealed that *FOXL2* functioned differently in the poGC and phGC, findings consistent with the those of our GO and KEGG enrichment analyses. *FOXL2* knockdown promoted cell proliferation and DNA replication, decreased cell apoptosis, and promoted mitosis by promoting the transition of poGC into the G2 phase, while none of these cell activities were affected by FOXL2-silencing in the phGC. It can be inferred that, *FOXL2* inhibits in poGC proliferation, probably by decreasing DNA replication and increasing apoptosis, and inhibits mitosis by regulating the cell cycle. Considering the fact that *FOXL2* is expressed at significantly higher levels in the poGC than in the phGC [14], it seems that *FOXL2* is of greater importance in regulating the functions of poGC. Since *FOXL2* silencing can promote apoptosis and proliferation of poGC, we hypothesized that *FOXL2* could inhibit premature ovulation of large follicles by preventing the over-proliferation of poGC. However, similar results were not found for the phGC. One reason might be that pre-hierarchical follicle growth was restricted; thus, the pathways associated with cell proliferation were blocked. *FOXL2* silencing was insufficient to activate these pathways; more factors were required. It seems that *FOXL2* plays a key role in follicular development. However, in follicular selection, it may participate but is not a key regulator.

In summary, our study indicated that a number of FOXL2-regulating genes play notable roles in the granulosa cell cycle and in proliferation and apoptosis through various related pathways, which should be further investigated in terms of follicular development.

## Supporting information

S1 Table Tequences of siRNA. (DOCX)

S2 Table Primers for qPCR. (DOCX)

S3 Table Statistics of the sequencing reads mapping to the reference genome. (DOCX)

S1 File (XLSX)

S2 File (XLSX)

S1 Raw Images (PDF)
