# Supplementary material for: Transcriptome sequencing revealed that knocking down FOXL2 affected cell proliferation, the cell cycle, and DNA replication in chicken pre-ovulatory follicle cells
Source: PLoS One. 2020 Jul 9;15(7):e0234795. doi: 10.1371/journal.pone.0234795 (PMC7347172; doi:10.1371/journal.pone.0234795)
Supplement: S1 Table — (DOCX) [file pone.0234795.s001.docx]

**S1 Table. sequences of siRNA**

| Gene Symbol | Primer Sequence (5’-3’) |
| --- | --- |
| FOXL2-siRNA | GCCUCAACGAGUGCUUCAUTT  AUGAAGCACUCGUUGAGGCTT |
| NC-siRNA | UUCUCCGAACGUGUCACGUTT  ACGUGACACGUUCGGAGAATT |
