# Supplementary material for: Transcriptome sequencing revealed that knocking down FOXL2 affected cell proliferation, the cell cycle, and DNA replication in chicken pre-ovulatory follicle cells
Source: PLoS One. 2020 Jul 9;15(7):e0234795. doi: 10.1371/journal.pone.0234795 (PMC7347172; doi:10.1371/journal.pone.0234795)
Supplement: S2 Table — (DOCX) [file pone.0234795.s002.docx]

**S2 Table. Primers for qPCR**

| Primer Name | Primer Sequence (5’-3’) | Product Size (bp) | Tm(℃) |
| --- | --- | --- | --- |
| GAPDH-F  GAPDH-R | GAGGGTAGTGAAGGCTGCTG  CACAACACGGTTGCTGTATC | 199 | 58 |
| FOXL2-F  FOXL2-R | TGTCCGGGATCTACCAGTAC  CTCGAACATGTCCTCGCA | 188 | 58 |
| ACTA2-F  ACTA2-R | TATTTTCCTGCCTTAAACTCA  AAGAGTTCAGAAATATGCAC | 168 | 58 |
| ALDH1A3-F  ALDH1A3-R | GTAGGGCTAATACAACTGACA  CATTTAGGGCTAGAATATCACCA | 176 | 58 |
| ASTN2-F  ASTN2-R | ATGAGTACACAGACACGGACCT  TCGTGTGTCCACCGCATACAGT | 200 | 58 |
| B3GALT5-F  B3GALT5-R | CCAGCTTTGTGATGAAAACTGAC  TCTTCAATCCTCACGTTCCAC | 166 | 58 |
| BFSP1-F  BFSP1-R | TGCCCGAACCTAGTGAACC  TTTTCCCCATTCAAATAGGTCT | 122 | 58 |
| BHMT-F  BHMT-R | ACCACTTGCTTTCCATACAC  AAATCCACAGCAGCCTCCAA | 166 | 58 |
| BMP3-F  BMP3-R | ATTGTGCCCGACGCTATCTCA  CCGACAGCTCTCACTATGCTCT | 175 | 58 |
| COL6A3-F  COL6A3-R | ATTTCCCACCTCGTAAGCAAC  ATGATCTTCTCTTTGGATCCGTA | 173 | 58 |
| COL8A1-F  COL8A1-R | GTTCAAATGCCATCCGAGCAG  AAAATGGTCCAAAACGGTCA | 200 | 58 |
| CSRP2-F  CSRP2-R | GCACAAGAACTGCTTCCGAT  TGCTCCCTGACCATAGCCAA | 142 | 58 |
| DIRAS2-F  DIRAS2-R | TTACTCTATCACCAGCCGACA  GGTCTCCATGAATGCACAC | 196 | 58 |
| EGFL7-F  EGFL7-R | GCAGATCCCTCCAGTGAAGT  AGTTGCTGGAGGGAGTGGGA | 173 | 58 |
| EML4-F  EML4-R | TGCCTTCTTGATCTAACAC  TTAGTCCGTTATTAAGTAGCC | 168 | 58 |
| FBLN5-F  FBLN5-R | CCGATCTTCTCCGTTACTCG  GGTCAAACCCATTAGTGCAT | 249 | 58 |
| FGF16-F  FGF16-R | ATTCAGGACTCTACCTCGGCAT  CCGTCCTTGTTCAGAGCCACGTA | 178 | 58 |
| FYB-F  FYB-R | AGCTGGATACACTAAAACCC  TTTCTTCACTCTCTTGTGCTT | 176 | 58 |
| GGT5-F  GGT5-R | CATCAGGCATCCTTGGCAAC  TTGCCACAACTGCCTTAGCTT | 136 | 58 |
| IGSF11-F  IGSF11-R | CCCTCCCAGCCTAGCCTCA  CTGCCCCACACTGGTCGTT | 162 | 58 |
| KCNK5-F  KCNK5-R | TGCCATCACTGGGAACAACACT  ATGTCAAGCACAGAGGAACTCCA | 170 | 58 |
| LFNG-F  LFNG-R | CCAGCATTCGCACCATTCCC  CTCCTCCCTCTTCTCCCCGTA | 128 | 58 |
| NR0B1-F  NR0B1-R | GCTCTTCAACCCGGATCTACCTG  CAAATCTGGCTTGGTCACCT | 125 | 58 |
| OPCML-F  OPCML-R | CGCGTCCACCTCATCGTGCAA  TGCACTCATACTCGCCCGACT | 226 | 58 |
| SCIN-F  SCIN-R | CTTACTCAGCAGACCCTCGT  GTGGTGCTTCCTCATACTCCA | 166 | 58 |
| SLC16A12-F  SLC16A12-R | GCGAATGCCTCTCTGTTCACC  ACGCTCAAAGCATAAGGCACT | 183 | 58 |
| SLC26A3-F  SLC26A3-R | GCAGTGTCCTTCCTTGATTT  GAAGAACATGGATGGCTTGA | 168 | 58 |
| SLC1A6-F  SLC1A6-R | GCGAACTCGATTATTTGTCCA  TCTGCATAACCCCATGTCGTA | 186 | 58 |
| SLC35F3-F  SLC35F3-R | CGCCCTTCACTCTAACGTGGT  CCTCTGCTTCGGAGACTGCT | 110 | 58 |
| SYTL2-F  SYTL2-R | ATCATGAACACGTATTACCAG  TTGAGATCTTCCAATGCACCA | 178 | 58 |
| TAGLN-F  TAGLN-R | ACATGTTCCAGACCGTTGACC  AGCCAATGATGTTCTTGCCCTC | 201 | 58 |
| TMEM72-F  TMEM72-R | TGCAGCTTCACTCCAACCAG  TTCAGTCTCGCTCTCCGCATC | 124 | 58 |
| TRIL-F  TRIL-R | CTGTTCCTCTGCAACCTGTC  CCAAAGCGGTCAAAGAGCAG | 131 | 58 |
| TYRP1-F  TYRP1-R | GGGCAAACACATTTATCACCA  TTGTATTCCCGGTTATGTCCA | 152 | 58 |
