# Supplementary material for: Transcriptome sequencing revealed that knocking down FOXL2 affected cell proliferation, the cell cycle, and DNA replication in chicken pre-ovulatory follicle cells
Source: PLoS One. 2020 Jul 9;15(7):e0234795. doi: 10.1371/journal.pone.0234795 (PMC7347172; doi:10.1371/journal.pone.0234795)
Supplement: S3 Table — (DOCX) [file pone.0234795.s003.docx]

**S3 Table. Statistics of the sequencing reads mapping to the reference genome.**

| Group | Sample-ID | Total Reads | Mapped Reads | Mapping rate | Clean reads | ≥Q30% |
| --- | --- | --- | --- | --- | --- | --- |
| poGC-KD | T1 | 70,837,046 | 59,730,612 | 84.32% | 35,418,523 | 92.16% |
|  | T2 | 67,424,554 | 57,748,459 | 85.65% | 33,712,277 | 92.53% |
|  | T3 | 83,069,318 | 70,998,316 | 85.47% | 41,534,659 | 92.44% |
|  | T4 | 68,496,780 | 58,315,623 | 85.14% | 34,248,390 | 92.56% |
| poGC-CT | T5 | 56,936,516 | 45,875,836 | 80.57% | 28,468,258 | 92.53% |
|  | T6 | 72,244,976 | 59,510,138 | 82.37% | 36,122,488 | 92.60% |
|  | T7 | 78,594,910 | 66,796,915 | 84.99% | 39,297,455 | 92.26% |
|  | T8 | 74,480,156 | 63,860,194 | 85.74% | 37,240,078 | 92.67% |
| phGC-KD | T9 | 62,409,956 | 54,531,223 | 87.38% | 31,204,978 | 92.65% |
|  | T10 | 43,307,336 | 36,257,862 | 83.72% | 21,653,668 | 92.24% |
|  | T11 | 48,788,854 | 42,516,863 | 87.14% | 24,394,427 | 90.88% |
|  | T12 | 47,655,894 | 41,115,729 | 86.28% | 23,827,947 | 90.71% |
| phGC-CT | T13 | 43,815,750 | 37,293,795 | 85.12% | 21,907,875 | 91.74% |
|  | T14 | 46,628,890 | 39,671,774 | 85.08% | 23,314,445 | 91.08% |
|  | T15 | 45,877,730 | 38,603,474 | 84.14% | 22,938,865 | 90.80% |
|  | T16 | 46,767,982 | 39,588,746 | 84.65% | 23,383,991 | 91.33% |
