# Supplementary material for: Transcriptome sequencing revealed that knocking down FOXL2 affected cell proliferation, the cell cycle, and DNA replication in chicken pre-ovulatory follicle cells
Source: PLoS One. 2020 Jul 9;15(7):e0234795. doi: 10.1371/journal.pone.0234795 (PMC7347172; doi:10.1371/journal.pone.0234795)
Supplement: S1 Raw Images — (PDF) [file pone.0234795.s006.pdf]

Table 1. Full-length gels and blots of western blot.

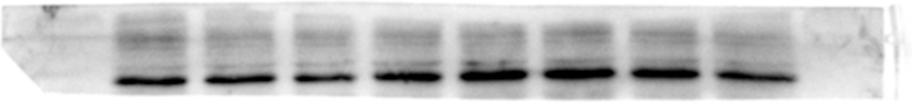

Fig. 1 PoGC cells transfected with *FOXL2*-siRNA or NC-siRNA, respectively, and the expression of FOXL2 protein detected by Western blot at 48 h post-transfection.

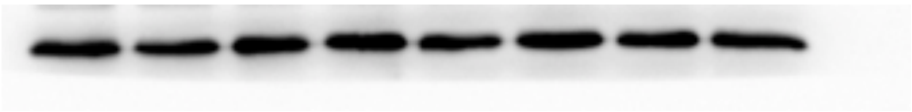

Fig. 2 PoGC cells transfected with *FOXL2*-siRNA or NC-siRNA, respectively, and the expression of GAPDH protein detected by Western blot at 48 h post-transfection.

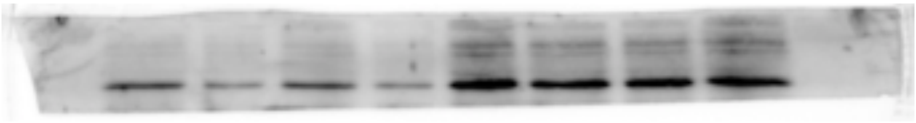

Fig. 3 PhGC cells transfected with *FOXL2*-siRNA or NC-siRNA, respectively, and the expression of FOXL2 protein detected by Western blot at 48 h post-transfection.

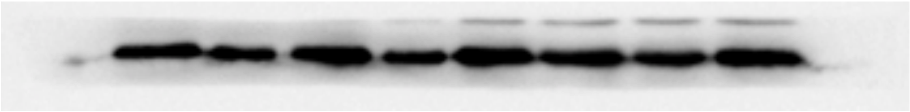

Fig. 4 PhGC cells transfected with *FOXL2*-siRNA or NC-siRNA, respectively, and the expression of GAPDH protein detected by Western blot at 48 h post-transfection.
